# *NFIA* Haploinsufficiency Is Associated with a CNS Malformation Syndrome and Urinary Tract Defects

**DOI:** 10.1371/journal.pgen.0030080

**Published:** 2007-05-25

**Authors:** Weining Lu, Fabiola Quintero-Rivera, Yanli Fan, Fowzan S Alkuraya, Diana J Donovan, Qiongchao Xi, Annick Turbe-Doan, Qing-Gang Li, Craig G Campbell, Alan L Shanske, Elliott H Sherr, Ayesha Ahmad, Roxana Peters, Benedict Rilliet, Paloma Parvex, Alexander G Bassuk, David J Harris, Heather Ferguson, Chantal Kelly, Christopher A Walsh, Richard M Gronostajski, Koenraad Devriendt, Anne Higgins, Azra H Ligon, Bradley J Quade, Cynthia C Morton, James F Gusella, Richard L Maas

**Affiliations:** 1 Genetics Division, Brigham and Women's Hospital and Harvard Medical School, Boston, Massachusetts, United States of America; 2 Renal Section, Boston University Medical Center, Boston, Massachusetts, United States of America; 3 Center for Human Genetic Research, Massachusetts General Hospital and Harvard Medical School, Boston, Massachusetts, United States of America; 4 Department of Pathology, Brigham and Women's Hospital and Harvard Medical School, Boston, Massachusetts, United States of America; 5 Division of Neurology, Children's Hospital of Western Ontario, London, Ontario, Canada; 6 Children's Hospital at Montefiore, Albert Einstein College of Medicine, Bronx, New York, United States of America; 7 Department of Neurology, University of California San Francisco, San Francisco, California, United States of America; 8 Division of Genetic and Metabolic Disorders, Department of Pediatrics, Wayne State University, Detroit, Michigan, United States of America; 9 Department of Neurosurgery, University Hospital, Geneva, Switzerland; 10 Department of Nephrology, University Hospital, Geneva, Switzerland; 11 Departments of Pediatrics and Neurology, Northwestern University Feinberg School of Medicine, Chicago, Illinois, United States of America; 12 Genetics Division, Children's Hospital Boston and Harvard Medical School, Boston, Massachusetts, United States of America; 13 Department of Obstetrics, Gynecology and Reproductive Biology, Brigham and Women's Hospital and Harvard Medical School, Boston, Massachusetts, United States of America; 14 Department of Neurology, Beth Israel Deaconess Medical Center and Harvard Medical School, Boston, Massachusetts, United States of America; 15 Howard Hughes Medical Institute, Beth Israel Deaconess Medical Center and Harvard Medical School, Boston, Massachusetts, United States of America; 16 Department of Biochemistry, State University of New York at Buffalo, Buffalo, New York, United States of America; 17 Centre for Human Genetics, University of Leuven, Leuven, Belgium; Medical Research Council Human Genetics Unit, United Kingdom

## Abstract

Complex central nervous system (CNS) malformations frequently coexist with other developmental abnormalities, but whether the associated defects share a common genetic basis is often unclear. We describe five individuals who share phenotypically related CNS malformations and in some cases urinary tract defects, and also haploinsufficiency for the *NFIA* transcription factor gene due to chromosomal translocation or deletion. Two individuals have balanced translocations that disrupt *NFIA*. A third individual and two half-siblings in an unrelated family have interstitial microdeletions that include *NFIA*. All five individuals exhibit similar CNS malformations consisting of a thin, hypoplastic, or absent corpus callosum, and hydrocephalus or ventriculomegaly. The majority of these individuals also exhibit Chiari type I malformation, tethered spinal cord, and urinary tract defects that include vesicoureteral reflux. Other genes are also broken or deleted in all five individuals, and may contribute to the phenotype. However, the only common genetic defect is *NFIA* haploinsufficiency. In addition, previous analyses of *Nfia^−/−^* knockout mice indicate that *Nfia* deficiency also results in hydrocephalus and agenesis of the corpus callosum. Further investigation of the mouse *Nfia* ^+/−^ and *Nfia* ^−/−^ phenotypes now reveals that, at reduced penetrance, *Nfia* is also required in a dosage-sensitive manner for ureteral and renal development. *Nfia* is expressed in the developing ureter and metanephric mesenchyme, and *Nfia* ^+/−^ and *Nfia* ^−/−^ mice exhibit abnormalities of the ureteropelvic and ureterovesical junctions, as well as bifid and megaureter. Collectively, the mouse *Nfia* mutant phenotype and the common features among these five human cases indicate that *NFIA* haploinsufficiency contributes to a novel human CNS malformation syndrome that can also include ureteral and renal defects.

## Author Summary

Central nervous system (CNS) and urinary tract abnormalities are common human malformations, but their variability and genetic complexity make it difficult to identify the responsible genes. Analysis of human chromosomal abnormalities associated with such disorders offers one approach to this problem. In five individuals described herein, a novel human syndrome that involves both CNS and urinary tract defects is associated with chromosomal disruption or deletion of *NFIA*, encoding a member of the Nuclear Factor I (NFI) family of transcription factors. This syndrome includes brain abnormalities (abnormal corpus callosum, hydrocephalus, ventriculomegaly, and Chiari type I malformation), spinal abnormalities (tethered spinal cord), and urinary tract abnormalities (vesicoureteral reflux). *Nfia* disruption in mice was already known to cause hydrocephalus and abnormal corpus callosum, and is now shown to exhibit renal defects and disturbed ureteral development. Other genes besides *NFIA* are also disrupted or deleted and may contribute to the observed phenotype. However, loss of one copy of *NFIA* is the only genetic defect common to all five patients. The authors thus provide evidence that genetic loss of *NFIA* contributes to a distinct CNS malformation syndrome with urinary tract defects of variable penetrance.

## Introduction

Complex human developmental phenotypes represent an especially difficult problem in human genetics. In many cases, congenital birth defects are believed to result from the combined effect of many genes, often with an environmental contribution, and frequently culminate in perinatal demise. Thus, for many cases, extended families do not exist, and approaches to disease gene identification based on linkage analysis are not possible. In addition, many developmental disorders are genetically heterogeneous, making the ascertainment of single contributory genes difficult.

The analysis of human balanced chromosome rearrangements offers a potential approach to this problem. Although unforeseen rearrangements and position effects may supervene [1,2], and a background rate of birth defects exists, human translocations provide powerful tools to identify genes that are essential to human development. Translocations may result in haploinsufficiency, the generation of fusion transcripts, or position effects, or act in combination with a second loss-of-function allele. The Developmental Genome Anatomy Project, DGAP (http://dgap.harvard.edu), has as its specific goal the ascertainment, recruitment, and analysis of individuals with chromosomal rearrangements and developmental disorders. A conspicuous class of such disorders is that involving the formation of the CNS and visceral organs.

Within the CNS, the corpus callosum is the largest interconnecting white matter tract in the brain, and it connects the association fibers of both hemispheres. Agenesis of the corpus callosum (ACC) is among the most common brain malformations in humans, with an incidence of 1 per 4,000 live births [3–5] and a prevalence as high as 3%–5% in individuals with neurodevelopmental disabilities [6,7]. In human embryos, the corpus callosum begins to develop at 11–12 wk gestation when the first fibers cross the midline to form the genu in the region of the commissural plate, and subsequent development proceeds from anterior to posterior, with formation of the anterior body, posterior body, and splenium, followed by a progressive enlargement that reflects the rapid expansion of the cerebral hemispheres [8]. Abnormalities of the corpus callosum can occur through a number of mechanisms, including defects in the genesis or survival of neuronal cells whose axons form the corpus callosum, and defects in axonal outgrowth, pathfinding, and midline crossing [9]. The etiology of ACC is thus heterogeneous and multifactorial, and both autosomal recessive and X-linked recessive mechanisms have been described [9] (see also Online Mendelian Inheritance in Man [OMIM, http://www.ncbi.nlm.nih.gov/entrez/query.fcgi?db=OMIM]). ACC is associated with certain chromosomal rearrangements [9] and occurs as a component of other genetic syndromes [10] and metabolic conditions [11], but its genetic heterogeneity and phenotypic pleiotropy have limited identification of the responsible genes to only a few of the more than 20 distinct loci that are associated with ACC, including one on the short arm of Chromosome 1 [12].

In addition to other CNS defects with which ACC is frequently associated, ACC may occur in conjunction with visceral organ malformations. For example, ACC and its associated brain and spinal cord lesions have been linked to vesicoureteral reflux (VUR), cystic kidney disease, renal agenesis and insufficiency, and neurogenic lower urinary tract dysfunction, a condition that includes neurogenic bladder and VUR [13–16]. Alternatively, single gene mutations may produce developmental defects in both the CNS and urinary tract. We show here that a novel human syndrome involving both CNS and urinary tract defects is associated with disruption or deletion of the *NFIA* gene at 1p31.3, which encodes a member of the Nuclear Factor I (NFI) family of transcription factors [17]. Disruption of *Nfia* in mice results in perinatal lethality, hydrocephalus, and ACC [18], and a recent study shows that *NFIA* controls the transition from neurogenesis to gliogenesis in the developing spinal cord [19]. Similar studies indicate that two other *Nfi* family members, *Nfib* and *Nfic*, are essential for lung, brain and tooth development [20,21]. However, the role that NFI transcription factors play in human disease has been unknown. Our results establish that *NFIA* haploinsufficiency is a likely contributor to a range of CNS defects, including ACC, hydrocephalus, ventriculomegaly, Chiari type I malformation, and tethered spinal cord, and that renal defects can also result from a disturbance in ureteral development.

## Results

### CNS and Urinary Tract Defects in Individuals with 1p31 Rearrangements

**Figure 1:**
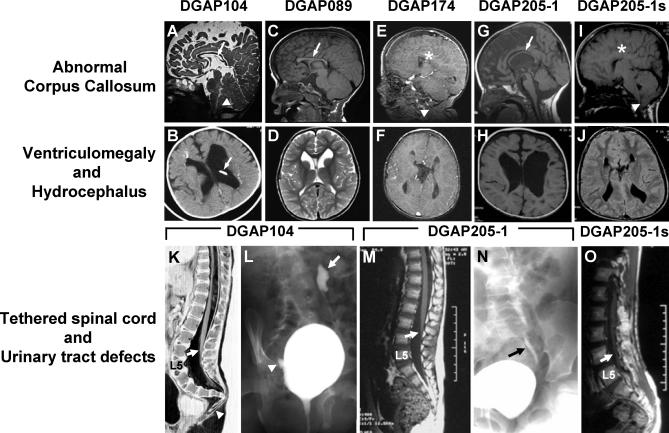
CNS and Urinary Tract Defects in Five Individuals with 1p31.3 Rearrangements (A–J) Brain CT or MRI show a thin corpus callosum in DGAP104 (arrow in A), DGAP089 (arrow in C), and DGAP205–1 (arrow in G); and agenesis of the corpus callosum in DGAP174 (asterisk in E) and DGAP205-1s (asterisk in I). Chiari type I malformation, a downward displacement of the tip of the cerebellar tonsils below the foramen magnum, was found in DGAP104 (arrowhead in A), DGAP174 (arrowhead in E), and DGAP205-1s (arrowhead in I). Congenital ventriculomegaly is present in DGAP089 (D), DGAP174 (F), and DGAP205-1s (J), and hydrocephalus was found in DGAP104 (B) and DGAP205–1 (H). An occipital shunt (arrow in B) was placed in DGAP104 to relieve severe hydrocephalus. (K) DGAP104 MRI shows a tethered spinal cord, with the extremity of the conus medullaris (arrow) at the level of the L4 vertebral body. Arrowhead shows a fishhook deformity of the lower sacral and coccygeal vertebrae. (L) VCUG of DGAP104 depicts left vesicoureteral reflux with retrograde tracking of dye through the ureter into the renal pelvis (arrow) and a right diverticulum at the ureterovesical junction (arrowhead). (M) Spine MRI of DGAP205–1 shows a tethered spinal cord with conus lying at the L3/L4 level (arrow). (N) VCUG of DGAP205–1 shows left vesicoureteral reflux (arrow). (O) Spine MRI of DGAP205-1s depicts a tethered spinal cord with conus lying at L5 (arrow).

We investigated five individuals enrolled in DGAP (i.e., DGAP104, 089, 174, 205–1, and 205–1s), each with a similar spectrum of CNS defects, and in three cases, of urinary tract defects. All five individuals had chromosomal rearrangements that variously involved 1p31. DGAP104 is a 6-y-old female diagnosed at birth with congenital hydrocephalus, a thin corpus callosum, Chiari I malformation, tethered spinal cord, and a low vertebral deformity (Figure 1A, 1B, and 1K). She was also found to have congenital bilateral dysplastic kidneys, and subsequently developed bilateral VUR, pyelonephritis, a ureterovesical junction diverticulum, and hydronephrosis, and required ureteral reimplantation surgery at age 2 y (Figure 1L; Tables 1 and S1). Chromosome analysis in the neonatal period revealed an apparent de novo balanced chromosome translocation between 1p31 and 20q13.

**Table 1:**
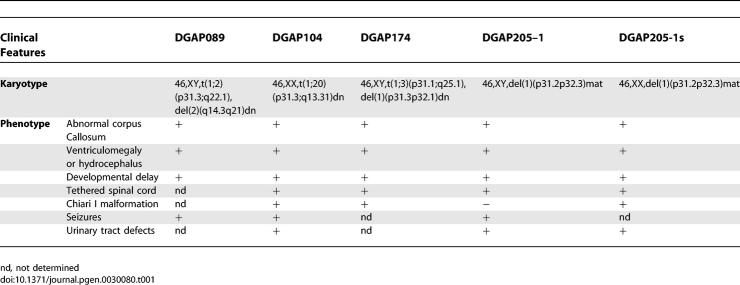
Common Clinical Features in Five Individuals with *NFIA* Haploinsufficiency

A second individual, DGAP089, is an 8-y-old male whose clinical profile was described previously (Table 1, S1) [22]. He has both an interstitial deletion on 2q and a balanced translocation involving 1p and 2q [22]. As an infant, he had poor fetal movement, and a CT scan revealed a vertical orientation of the ventricles consistent with a primary defect of the corpus callosum. At age 2 y, a CT scan revealed ventriculomegaly, and at age 6.5 y a brain MRI showed a hypoplastic corpus callosum, nonprogressive ventriculomegaly, and a gray matter heterotopia (Figure 1C and 1D; Tables 1 and S1). Renal ultrasound revealed no major abnormalities, but a definitive evaluation for urinary reflux (i.e., a voiding cystourethrogram, or VCUG) was not performed.

A third individual, DGAP174, who exhibited complete ACC and enlarged ventricles by second trimester ultrasound, had both a de novo translocation of 1p31.1 and 3q25.1 and an interstitial deletion of 1p31. A postnatal brain CT scan confirmed these findings and also revealed ventriculomegaly and a tethered spinal cord. A brain MRI at age 3 y revealed a Chiari type I malformation and dysplasia of the anterior left temporal fossa, in addition to ventriculomegaly and ACC (Figure 1E and 1F; Tables 1 and S1). As with DGAP089, no major renal abnormalities were detected by ultrasound, and VCUG was not performed.

Lastly, we ascertained two previously described half-siblings who shared CNS and renal phenotypes similar to those in DGAP104 [23]. Both individuals, DGAP205–1 and DGAP205-1s, have a maternally inherited, unbalanced interstitial microdeletion, del(1)(p31.3p32.3), and exhibited an abnormal corpus callosum, congenital hydrocephalus, syringomyelia, tethered spinal cord, and urinary tract phenotypes including VUR (Figure 1G–1J and 1M–1O) [23].

### Cytogenetic and Genetic Analyses of the Chromosomal Rearrangements

**Figure 2:**
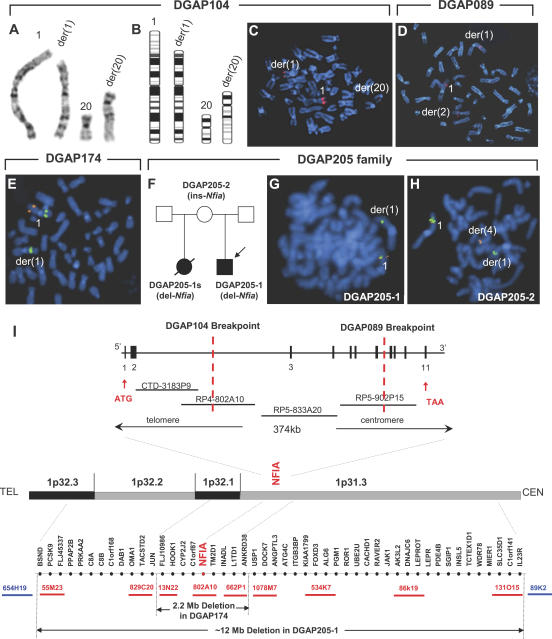
*NFIA* is Deleted or Disrupted in All Five Individuals with 1p31.3 Rearrangements (A and B) Partial karyogram (A) and ideogram (B) of DGAP104 show the chromosome translocation t(1;20)(p31.3;q13.31)dn. (C) FISH analysis of DGAP104; BAC RP4-802A10 (red signals) hybridizes to the normal Chromosome 1 and der(1) and der(20) chromosomes, thus spanning the 1p31.3 breakpoint. (D) FISH analysis of DGAP089 depicts BAC RP5-902P15 (red signals) hybridization to the normal Chromosome 1 and der(1) and der(2) chromosomes, thus spanning the 1p31.3 breakpoint. (E) FISH analysis reveals that BAC RP5-902P15 (overlapping with *NFIA,* orange color) is deleted from the der(1) in DGAP174. The nondeleted BAC RP11-134C1 (green) is present on both the normal and derivative Chromosome 1. (F) DGAP205 pedigree with *NFIA* deletion (arrow indicates the DGAP205–1 proband). DGAP205–1 and half-sister DGAP205-1s have an unbalanced interstitial microdeletion, del(1)(p31.3p32.3), while their phenotypically normal mother DGAP205–2 has a balanced chromosome rearrangement due to an insertion of 1p31.3-p32.3 into Chromosome 4, ins(4;1)(q35;p31.3p32.3). del-*NFIA,* deletion of *NFIA*; ins-*NFIA,* insertion of *NFIA*. (G and H) FISH analyses show that BAC RP5-902P15 (overlapping with *NFIA,* orange color) is absent from the der(1) in DGAP205–1 (G) and der (1) in the mother DGAP205–2 (H), but present in the der(4) in the latter (H). The nondeleted BAC RP4-654H19 (green) is present on both the normal and derivative Chromosome 1. (I) *NFIA* exon–intron structure is shown in the upper part with select exons numbered, and the relevant BAC contig below. Locations of the 1p31.3 translocation breakpoints in DGAP104 and DGAP089 are indicated by red dotted vertical lines. The lower part of (I) depicts 1p31.3–1p32.3 genomic regions with the cytogenetic bands on the short arm of Chromosome 1. TEL represents telomeric orientation, and CEN represents centromeric orientation. Known genes in this region are represented by dots and gene names. FISH-verified BAC clones are represented by horizontal bars. The full names of the BAC clones are listed in Materials and Methods. A 2.2-Mb genomic region deleted in DGAP174 and a ~12-Mb genomic region deleted in DGAP205–1 and 205–1s are shown. Deleted BAC clones tested by FISH are designated in red and nondeleted clones in blue. BAC 802A10 overlaps with *NFIA* and is deleted.

To determine whether a common genetic defect underlay the phenotypes of these individuals, we analyzed the 1p31 region in all five patients and found that the *NFIA* gene was either disrupted or deleted in each case. DGAP104 has a de novo balanced translocation, 46,XX,t(1;20)(p31.3;q13.31)dn (Figure 2A, 2B). By metaphase fluorescence in situ hybridization (FISH), we identified a bacterial artificial chromosome (BAC) clone (RP4-802A10) that mapped to 1p31.3 and hybridized to the breakpoints of the der(1) and der(20) chromosomes (Figure 2C). The translocation breakpoint at 1p31.3 disrupts intron 2 of *NFIA*, which is composed of 11 exons and spans ~374 kb of genomic DNA (Figure 2I). This result was confirmed by Southern blot analysis (Figure S1).

In addition to disruption of *NFIA* at 1p31.3 in DGAP104, *C20orf32* was also disrupted by the 20q13.31 breakpoint. A contribution of *C20orf32* disruption to the spinal and kidney phenotypes in DGAP104 is unlikely, however, because *C20orf32* expression was not detected in the developing spinal cord or kidney by in situ hybridization (Figure S2). By array comparative genomic hybridization (aCGH), we excluded any additional chromosome abnormalities in DGAP104 at ~1 Mb resolution.

For DGAP089, sequential FISH led to identification of a 1p31.3 breakpoint-spanning BAC, RP5-902P15, which hybridized to Chromosome 1 and to both der(1) and der(2) chromosomes (Figure 2D). Subsequent FISH and Southern blot analyses (Figure S3) refined the breakpoint to ~3.9 kb between exons 7 and 8 of *NFIA* (Figure 2I)*.* Similar analyses of the 2q breakpoint revealed a split signal for BAC RP11-745P9, thus localizing the breakpoint to ~138 kb in 2q22.1, which contains no annotated genes. However, metaphase FISH followed by aCGH at ~1 Mb resolution revealed a ~12-Mb interstitial deletion in 2q proximal to the 2q translocation breakpoint, for which the karyotype is 46,XY,t(1;2)(p31.3;q22.1),del(2)(q14.3q21)dn. The 39 genes within this deletion interval may thus also contribute to the DGAP089 phenotype [22].

To establish further whether disruption of *NFIA* is primarily associated with the congenital CNS anomalies observed in DGAP104 and DGAP089, we next investigated DGAP174, who has both a t(1;3)(p31.1;q25.1)dn and an interstitial deletion, del(1)(p31.3p32.1)dn. The 1p31.1 and 3q25.1 translocation breakpoints were refined to 150 and 180 kb, respectively. The only other potentially relevant gene in these intervals is *NEGR1*, which is disrupted by the 1p31.1 breakpoint. In rat, NEGR1 protein is expressed only after E16, with peak expression occurring postnatally, after corpus callosum formation [24]. However, by RT-PCR, *NEGR1* transcript is expressed in human cerebral cortex, hippocampus, corpus callosum, and cerebellum (unpublished data); hence, a contribution to the DGAP174 CNS phenotype is possible. On the other hand, the chromosome deletion in DGAP174, which was delimited by FISH and aCGH to 2.2 Mb at 1p31.3–1p32.1 (Figures 2E and S4), also results in the complete deletion of *NFIA* and of eight additional genes (Figure 2I).

Lastly, we performed metaphase FISH analyses on chromosomes isolated from the two half-siblings DGAP205–1 and DGAP205-1s and their mother DGAP205–2 (Figure 2F–2I). The 1p31.3–1p32.3 region, encompassing ~12 Mb and containing the entire *NFIA* gene and ~47 additional genes, is deleted in both DGAP205–1 and DGAP205–2 (Figure 2G–2I). The mother, DGAP205–2, is phenotypically normal but has an apparent balanced rearrangement in which 1p31.3p32.3 is inserted into Chromosome 4 with no loss of genetic material, and her karyotype is therefore designated 46,XX,ins(4;1)(q35;p31.3p32.3) (Figure 2H) [23].

### *NFIA* Haploinsufficiency Is Common to All Five Cases

All five individuals share strikingly similar CNS phenotypes, including abnormalities of the corpus callosum, hydrocephalus, and ventriculomegaly. All also share disruption or deletion of *NFIA,* and in each case, the nonrearranged or nondeleted *NFIA* allele was subjected to DNA sequencing and no mutations were identified (unpublished data). Therefore, all five cases have *NFIA* haploinsufficiency in common. *NFIA* is highly expressed in multiple regions of the human brain, including the embryonic and adult corpus callosum (Figure S5) [25], and ACC and hydrocephalus were observed in *Nfia* ^−/−^ mutant mice [18].

In each DGAP case, one or more additional genes were also directly affected as a consequence of either the translocation or deletion. These additional genes may contribute to or modify the nature of the phenotype attributable to *NFIA* disruption or deletion. However, the identities of these genes differ among the five DGAP cases, except for 205–1 and 205–1s, who share the same 12-Mb deletion; and 205–1, 205–1s, and 174, who share a common 2.2-Mb deletion region (Figure 2I). *C20orf32* is disrupted in addition to *NFIA* in DGAP104*,* whereas in DGAP089 disruption of *NEGR1* and deletion of 39 genes in del(2)(q14.3q21) occurred. Besides the disruption or deletion of *NFIA,* none of the other of these genetic aberrations is shared by more than three cases. Therefore, the most parsimonious explanation for the observed CNS phenotypes is *NFIA* haploinsufficiency, which is the only common genetic defect shared by all five individuals.

To test whether intragenic mutations in *NFIA* are associated with abnormal callosal development and other CNS phenotypes, we sequenced the 11 exons and intron-exon boundaries of *NFIA* in 84 patients with various combinations of syndromic CNS phenotypes, including abnormal corpus callosum, tethered spinal cord, Chiari I malformation, hydrocephalus, and urinary tract defects (Table S2). Sequence analysis was also performed on a group of 96 individuals that included both syndromic and nonsyndromic ACC, and on another group of 39 individuals with nonsyndromic tethered cord syndrome. Although several known SNPs were detected, no intragenic mutations were identified. Thus, intragenic mutation in *NFIA* is not a frequent cause of the CNS defects described here.

### Further Analysis of Mouse *Nfia* Mutants Reveals Additional Phenotypes

**Figure 3:**
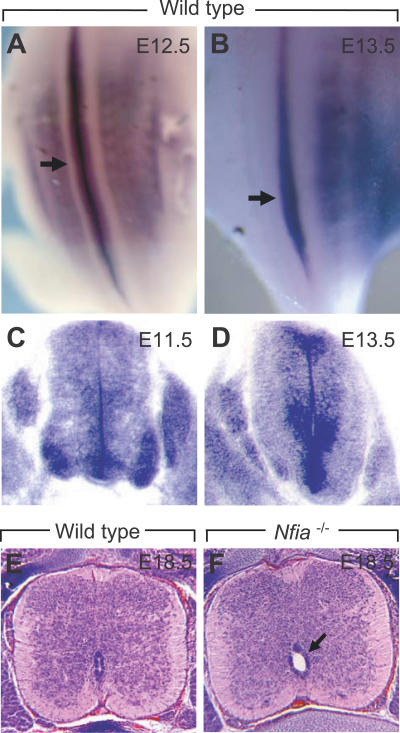
*Nfia* Spinal Cord Expression and Syringomyelia Phenotype in *Nfia* ^−/−^ Mice (A and B) Whole mount in situ hybridization shows abundant *Nfia* expression in the spinal cord (arrow) of E12.5 (A) and E13.5 (B) mouse embryos. The expression maximum (arrow) transits from rostral to caudal as development proceeds. (C and D) *Nfia* expression is restricted to the floor plate and spinal ganglia at E11.5 (C), and to the ventral spinal cord at E13.5 (D). (E and F) Spinal cord phenotype in *Nfia* E18.5 embryos. (E) The lumbar spinal cord central canal is nearly obliterated in wild-type embryos, whereas (F) shows persistent dilation of the lumbar spinal cord central canal (syringomyelia, arrow) in E18.5 *Nfia* ^−/−^ mutants.

To gain further evidence for the assignment of *NFIA* as the gene responsible for the CNS and urinary tract defects observed in the five individuals, we re-investigated the previously described *Nfia* ^−/−^ knockout mouse [18]. Because syringomyelia, a persistent patency of the central canal within the spinal cord, is often associated with Chiari type I malformation [26] and was observed in DGAP205-1s but not previously noted in *Nfia* ^−/−^ mice [18], we first examined *Nfia* expression in the developing spinal cord (Figure 3A–3D). From E11.5–13.5, the expression of *Nfia* in the spinal cord moves dynamically from rostral to caudal as development proceeds, coincident with the timing of central canal closure (Figure 3A, 3B). Moreover, *Nfia* expression resides in the basal ventricular zone surrounding the central canal (Figure 3C, 3D). Four of six (66%) *Nfia* ^−/−^ newborns exhibited syringomyelia that was manifest as an enlarged central canal and principally confined to the lumbar region (Figure 3E, 3F). Syringomyelia has been proposed to result from progressive, mechanical overload and dissection of the rostral spinal cord by elevated cerebral spinal fluid hydrostatic pressure such as that associated with hydrocephalus [27]. Indeed, all five individuals with *NFIA* haploinsufficiency exhibited hydrocephalus or ventriculomegaly. However, mouse *Nfia* expression in the spinal cord surrounds the central canal and defective canal closure in *Nfia* ^−/−^ mutants precedes the development of hydrocephalus. This suggests that syringomyelia may result from a developmental defect in the dynamic process of canal closure.

### *Nfia* Expression and Abnormal Kidney Development in *Nfia* Mutants

DGAP104, 205–1 and 205–1s share similar urinary tract phenotypes. Although the CNS defects in all five individuals are consistent with findings in mouse *Nfia* mutants [18], it remained possible that the urinary tract defects might result from the genetic lesion at 20q13.31 (in DGAP104), from haploinsufficiency for other genes in the 1p31.3-p32.3 deletion interval (in DGAP205–1 and DGAP205-1s), or from incidental mutations in other loci. Previously, it was noted that *Nfia* homozygotes died shortly after birth [18]. Based upon prior experience with mouse mutants with renal agenesis and dysplasia [28–32], we hypothesized that some part of this perinatal lethality might be explained by defects in renal development.

**Figure 4:**
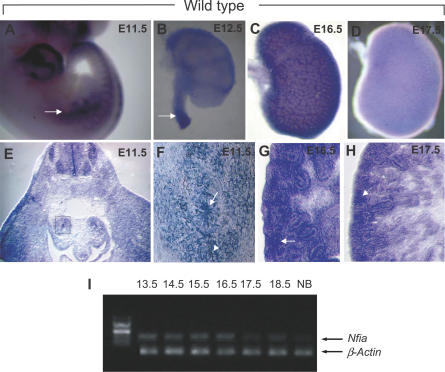
*Nfia* Expression in the Developing Mouse Urinary Tract (A–D) Whole mount in situ hybridization shows *Nfia* expression in the developing nephric duct (arrow in A), ureter (arrow in B), and metanephros between E9.5 (not shown) and E16.5, becoming downregulated at E17.5. Reticular pattern of *Nfia* expression is in the kidney from E14.5–16.5 (C). (E–H) Section in situ hybridizations shows *Nfia* expression in the ureteral epithelium (arrow in F) and surrounding mesenchyme (arrowhead in F) at E11.5 (F is the enlarged view of boxed region in E), and in stromal mesenchyme (arrows in G and H) at E16.5–17.5. (I) RT-PCR of the developing mouse kidney showing that *Nfia* is abundantly expressed up to E16.5 and begins to be downregulated at E17.5. β-actin is used as RNA loading control. NB, newborn.

To determine whether the urinary tract phenotypes in DGAP104, 205–1, and 205–1s were linked to *NFIA* disruption, we investigated *Nfia* expression in the developing murine kidney and renal morphology in *Nfia* ^−/−^ mice. By in situ hybridization, *Nfia* was abundantly expressed in the developing nephric duct and metanephros from E9.5 to E16.5, correlating with the stages of ureteric bud outgrowth, metanephric induction, and rapid nephron morphogenesis; expression began to be downregulated at E17.5 (Figure 4A–4I). At E9.5, *Nfia* expression appeared in the nephric duct and persisted at E10.5 and 11.5 in the developing ureter, becoming more restricted to the distal ureter by E12.5 (Figure 4A and 4B). *Nfia* was expressed in both ureteric bud epithelium and the surrounding mesenchyme at these time points (Figure 4E and 4F). By E16.5, *Nfia* expression became restricted to stromal mesenchyme, and was downregulated a day later (Figure 4C, 4D, 4G, and 4H).

**Figure 5:**
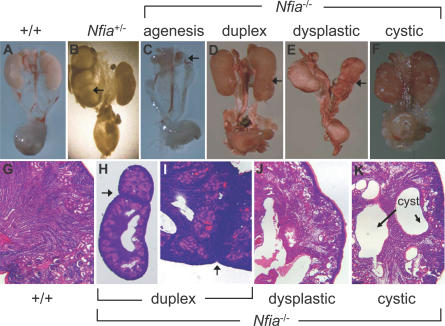
Spectrum of Kidney Phenotypes in *Nfia* ^−/−^ Newborn Mice Whole mounts of dissected urinary tracts from newborn mice of the indicated genotype (A–F). Normal wild-type newborn kidney and renal histology (A and G); (B) rare *Nfia* ^+/−^ showing hydronephrosis (arrow); (C–F and H–K) *Nfia* ^−/−^ mutants showing bilateral renal agenesis (arrow in C), an elongated, partial duplex kidney with the abnormal cortical zone that demarcates the two poles shown with an arrow (D, H, and I), severely dysplastic kidney with irregular renal surface (arrow in E) and disorganized renal parenchyma (E and J), and nodular *Nfia* homozygous newborn kidney (F) with renal tubule cystic dilatation (K).

We next examined the kidney morphology of *Nfia* ^−/−^ newborn mice. Remarkably, 13 of 19 (68%) *Nfia* ^−/−^ newborns displayed agenesis, dysplastic, cystic, or duplex kidneys (Figure 5A–5K). A small number of *Nfia* ^−/−^ mutants exhibited a partial duplex kidney phenotype. This was apparent from both the elongated kidney morphology with a central constriction, and from histological evidence of an ectopic nephrogenic zone that demarcated discrete rostral and caudal nephric poles (Figure 5D, 5H, and 5I).

The presence of kidney defects was not confined to *Nfia* ^−/−^ mutants. Four of 18 (22%) of *Nfia* ^+/−^ newborn mice also expressed hydronephrosis (Figure 5B). The possibility that these *Nfia* ^+/−^ newborns might be *Nfia* ^−/−^ mice that were misgenotyped was excluded because the *Nfia* ^+/−^ affecteds were offspring of an *Nfia* ^+/−^ × *Nfia* ^+/+^ cross. The sensitivity of mouse kidney development to *Nfia* gene dosage supports the conclusion that kidney defects can occur in humans carrying disruption or loss of a single *NFIA* allele. Therefore, disruption or deletion of *NFIA* in DGAP104, 205–1, and 205–1s likely explains the kidney phenotypes observed in these individuals.

We next sought to characterize further the renal defects in *Nfia* ^−/−^ embryos by marker experiments. Markers analyzed included Wt1 (glomerular podocytes), DBA (distal tubule and collecting duct), LTL (proximal tubule), and E-cadherin (cell adhesion and aggregation). No consistent changes from wild type that would reflect a deficiency of a particular cell type were observed (unpublished data). These results suggest that the renal defects in *Nfia* mutants are not lineage- or segment-specific.

### *Nfia* Kidney Defects Involve Defective Ureteral Development

The relative preservation of tubular and glomerular markers in *Nfia* mutant kidneys, the diverse spectrum of renal abnormalities, and the presence of hydronephrosis suggested that the renal parenchymal changes might be secondary to ureteral reflux or obstruction. To test this hypothesis, we crossed a *Hoxb7* promoter-directed *GFP* reporter allele into the *Nfia* ^+/−^ and *Nfia* ^−/−^ backgrounds. The *Hoxb7-GFP* transgene is specifically expressed in ureteral epithelium as early as E10.5, and thereafter in the ureteric bud and its derivatives, and eventually in renal tubular epithelium [33].

**Figure 6:**
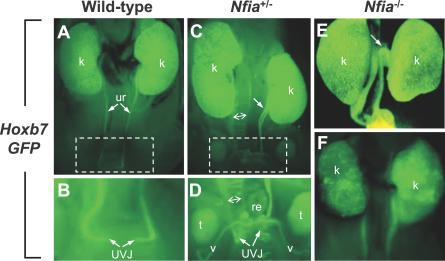
*Nfia* is Required for Normal Ureteral Development A *Hoxb7-GFP* transgenic reporter in the *Nfia* ^+/−^ and *Nfia* ^−/−^ backgrounds reveals ureteral defects in newborn mice. (A–D) Wild-type and *Nfia* ^+/−^ newborns with megaureter (with extent of dilation indicated by two-headed arrow) (A, C) and partial ureteral duplication (arrow) in the mutant (C). Higher power views of boxed areas in (A) and (C), showing distortion of the mutant ureter at the UVJ (B and D); note that the vas deferens in the *Nfia* ^+/−^ male (C) is also GFP–positive. (E and F) Abnormalities in *Nfia* ^−/−^ newborns include proximal flexion of the ureter (E) and dysplastic kidney (F). k, kidney; t, testis; ur, ureter; UVJ, ureterovesical junction; v, vas deferens

Remarkably, when assayed by GFP expression, four of 20 (20%) *Nfia* ^+/−^ and two of six (33%) *Nfia* ^−/−^ newborns exhibited clear abnormalities of ureteral development (Figure 6A–6F). These abnormalities fell into three classes. First, we observed a partial duplication of the ureter (Figure 6C), which correlates with the duplex kidney phenotype. A second class consisted of mutant ureters that were dilated, either as a consequence of obstruction or from reflux (Figure 6C and 6D). In the third class, we observed abnormal flexure of the rostral ureter, with the site of flexion near the renal pelvis (Figure 6E), which could lead to obstruction. To determine whether prolonged reflux and obstruction in *Nfia* ^−/−^ mutants affected postnatal kidney development, we analyzed two rare *Nfia* ^−/−^ postnatal survivors. Both P16 *Nfia* ^−/−^ developed severe hydronephrosis, whereas kidneys in their wild-type littermates were normal (Figure S6).

**Figure 7:**
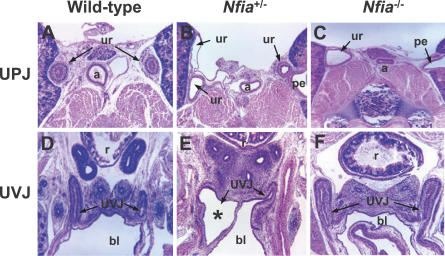
Ureter Defects at UPJ and UVJ in *Nfia* Mutant Mice (A–C) Hematoxylin and eosin histology depicts duplication and dilatation of ureter at the UPJ in *Nfia* ^+/−^ (B, 60X) and *Nfia* ^−/−^ (C, 60X) newborn mice. (D–F) Hematoxylin and eosin staining shows dilatation of UVJ in some *Nfia* ^+/−^ mutant mice (* in E, 60X); the majority of *Nfia* ^+/−^ and *Nfia* ^−/−^ mice show a normal UVJ (F, 60X). a, abdominal aorta; bl, bladder; pe, pelvis; r, rectum; ur, ureter; UVJ, ureterovesical junction

Lastly, we analyzed the histology of two key ureteral structures, the ureteropelvic junction (UPJ), which connects the ureter to the kidney, and the ureterovesical junction (UVJ), which connects the ureter to the bladder. Both UPJ and UVJ histological defects were noted in *Nfia* ^+/−^ newborns, while only UPJ defects were identified in *Nfia* ^−/−^ newborns (Figure 7A–7F). The presence of UPJ and UVJ dilation in *Nfia* mutant mice (Figure 7B, 7C, and 7E) is consistent with the observations of VUR and hydronephrosis in DGAP104, 205–1, and 205–1s. Thus, abnormalities in ureteral development comprise a significant part of the *Nfia* mutant phenotype. Moreover, because proper formation of the vertebrate kidney depends upon induction by the ureteric bud, ureteral abnormalities could account for aspects of the kidney defects in *NFIA* haploinsufficient patients and mutant mice.

## Discussion

### *NFIA* Haploinsufficiency as a Pathogenetic Mechanism

The five individuals studied here share *NFIA* haploinsufficiency caused by translocation (DGAP089 and 104) or deletion (DGAP174, 205–1, and 205–1s). All five also share abnormalities of the corpus callosum, and partly share other CNS phenotypes, including ventriculomegaly, congenital hydrocephalus, Chiari type I malformation, and tethered spinal cord. All five individuals also have developmental delay and three exhibited seizure disorders. Three of the five also have urinary tract defects, including VUR. Prior work established that *Nfia* loss of function in the mouse results in ACC and abnormal development of the hippocampal commissure, two major axonal tracts that connect the cerebral hemispheres, and an associated hydrocephalus that develops in rare postnatal survivors [18,34]. We also found that the mouse *Nfia* mutant recapitulates the VUR phenotype in these humans. Therefore, although other affected genes may contribute to the overall phenotype, these cases suggest that *NFIA* haploinsufficiency can account for the observed CNS and renal defects.

It is important to acknowledge that a contribution to the developmental phenotypes identified here from additional genes that reside within various deletion intervals, or that also suffer disruption by breakpoints, is not excluded. In all five cases, additional genes besides *NFIA* are also disrupted or deleted, so that in no single case is a defect in *NFIA* the only genetic abnormality. While DGAP104 only inactivates *NFIA* and *C20orf32* and the latter is an unlikely contributory factor, the most extreme cases are DGAP089 and the DGAP205 half-siblings, which contain deletions that involve 39 and 47 genes, respectively. Because these two deletions involve different chromosomes, none of the deleted genes are shared. However, in both cases, many more genes are affected than just *NFIA*, and some may participate in the observed phenotypes. For example, two cases described in the literature report 2q deletions that overlap with the del(2)(q14.3q21) in DGAP089, and these also involve ACC [35,36]. Therefore, in the absence of intragenic mutations in *NFIA,* the definitive argument that *NFIA* is the gene responsible for the CNS and renal phenotypes in these five patients cannot be made.

The inability to identify intragenic mutations in *NFIA* in cases involving ACC, hydrocephalus, tethered cord syndrome, and urinary tract defects could suggest that the phenotype of heterozygous intragenic loss-of-function *NFIA* mutations might differ from that described here. Indeed, as noted, it is quite plausible that in any individual DGAP case, the observed phenotype represents the additive effect of *NFIA* haploinsufficiency plus other loci that are deleted or disrupted. On the other hand, at least 20 discrete loci have been implicated in ACC alone, so that the failure to detect intragenic mutations in *NFIA* is not surprising. Ultimately, formal definition of the *NFIA* hemizygous loss-of-function phenotype would be strengthened by identification of intragenic loss-of-function *NFIA* mutations.

Lastly, it is well recognized that both chromosomal translocations and deletions may engender position effects that alter gene expression at considerable distances from the site of a chromosomal aberration (reviewed in [1,2]). For example, *Shh* expression in the limb bud mesenchyme is controlled by a regulatory region located ~1 Mb upstream within the unrelated *Lmbr1* gene [37]. Position effects on neighboring genes for mouse knockouts have been described [38]. The mouse *Nfia* mutant results from a small exon 2 deletion, yet still accurately recapitulates many features of the human phenotype. Therefore, one would have to posit the existence of a conserved regulatory element within the exon 2 deletion region that would act on genes 3′ to *Nfia*, which exhibit conservation of synteny between mouse and human. However, the genes immediately neighboring *NFIA* are not known to play a role in CNS or kidney development. These include *C1orf87* (GeneID 127795) and *TM2D1* (beta amyloid binding protein, GeneID 83941), which reside approximately 1 Mb 5′ and 200 kb 3′ of *NFIA*, respectively. Additional genes that reside at larger distances from *NFIA* might be affected by a position effect, but none are obvious candidates. Taking these factors into consideration, we conclude that a true position effect is unlikely to explain the observed phenotypes.

### Nature of the CNS Phenotype

Formation of the corpus callosum causes inversion of the cingulate gyri, which gives the medial surface of the brain its characteristic pattern. In ACC, the cingulum remains everted at sites of agenesis, and the sulci of the medial brain extend into the third ventricle. The findings in DGAP174 of an everted cingulate gyrus and longitudinal bundles of Probst are consistent with primary dysgenesis of the corpus callosum. Three midline populations contribute to formation of the corpus callosum: the glial sling, the glial wedge, and glia within the indusium griseum and its precallosal extension, the hippocampal continuation [39–41]. *NFIA* protein is expressed in all three midline populations, which fail to develop properly in *Nfia* ^−/−^ mice [34]. These populations normally form the corticoseptal boundary that prevents callosal axons from entering the septum.

The function of *NFIA* in formation of these neuronal populations places it within the class of genes that regulate axonal midline crossing. The prototypical regulatory gene in this class is *roundabout* or *robo,* which was originally identified in *Drosophila*. *Roundabout* encodes a transmembrane receptor expressed by migratory axons after they cross the CNS midline. Robo binds the extracellular ligand, Slit, which is expressed by midline glia and functions as a chemorepulsive cue that prevents axons from midline recrossing. This function extends to mammals, as mice lacking *Robo1* or *Robo2* exhibit CNS phenotypes that include abnormal midline commissural axonal guidance, and *Robo1* mutants in particular exhibit callosal dysgenesis [42,43]. Similarly, Slit ligands also play a role in callosal development. *Slit2* mutants display a small corpus callosum with a reduced number of traversing axons [44–46], while *Slit2* glial expression during callosal development in *Nfia* mutants is reduced [34].

Interestingly, *Nfia*, *Robo2*, and *Slit2* mouse mutants share not only axonal midline crossing defects, but also phenotypically related renal and ureteral defects. Mice deficient for *Robo2* or *Slit2* exhibit duplex kidney and megaureter phenotypes [32,47] that in some ways resemble those in *Nfia* mutants. Our recent study also implicated ROBO2 signaling in the pathogenesis of a subset of human VUR [32]. These related phenotypes raise the possibility that *Nfia* and SLIT–ROBO signaling are functionally linked in both CNS and ureteral development.

### Relationship between Kidney and Ureter Phenotypes

Based on *Nfia* expression in the developing kidney and the presence of kidney hypoplasia in DGAP104, we identified several distinct kidney phenotypes, including renal dysplasia and hydronephrosis in *Nfia* mutant mice. Hydronephrosis usually results from an obstruction in the flow of urine at the level of the UPJ or UVJ. This results in an obstructive uropathy in which back pressure from the accumulation of urine in the ureter and renal pelvis results in destruction and distortion of the renal parenchyma. The presence of hydronephrosis in *Nfia* ^−/−^ and *Nfia* ^+/−^ mutants therefore suggests that the observed renal defects reflect a primary disturbance in ureteral development.

A striking finding in *Nfia* mutant embryos and newborns is the presence of clear ureteral abnormalities: megaureter, abnormal ureteral folding, abnormalities at the UVJ and UPJ, and in a small number of cases, partial duplication of the ureter. These findings are consistent with the strong expression of *Nfia* in the developing nephric duct and ureter at E9.5–13.5. The ureteral duplication phenotype, distinct from normal patterns of ureteric bud branching [48], very likely explains the finding of a partial duplex kidney. Because ureteric bud contact with uninduced metanephric mesenchyme triggers the inductive cascade [49], contact by two separate ureteral branches should produce a partial duplex kidney. In addition to abnormal ureteral development, the VUR in DGAP104 and in the DGAP205 half-siblings may also develop as a consequence of the tethered spinal cord defect [50,51].

In DGAP089 and 174, renal ultrasound revealed no major abnormalities and urinary reflux was not observed. However, subtle anatomic defects in the ureter or kidney are often sub-clinical, and may exist below the limit of detection. In addition, these results are consistent with those in *Nfia^+/−^* and *Nfia* ^−/−^ mutants, where the penetrance of overt kidney or ureteral defects was only 22% and 68%, respectively. One explanation for the incomplete penetrance of ureteral or kidney defects in *Nfia* mutants could be functional redundancy with *Nfib*. In mice, *Nfib* is strongly expressed in the developing nephric duct, kidney, and ureter at E10.5–11.5, where its expression overlaps with that of *Nfia* (unpublished data). The other two Nuclear Factor I family members, *Nfic* and *Nfix*, are expressed only at lower levels in the developing ureter and kidney. In addition, *Nfib* ^−/−^ mice exhibit callosal agenesis and forebrain defects similar to those seen in *Nfia* ^−/−^ mice [21]. Thus, partial redundancy may exist between *Nfia* and *Nfib* in both CNS and urinary tract development. Genetic combinations of mutant alleles for *Nfia* and *Nfib* will be required to address this question definitively, and to further disclose the roles of Nfi factors in development.

In sum, our results suggest that *NFIA* haploinsufficiency in humans results in a thin, hypoplastic or absent corpus callosum, and define the spectrum of defects attributable to *NFIA* loss of function to include additional CNS and urinary tract defects that were not previously apparent in the *Nfia* mouse mutant. These results illustrate the powerful synergy that occurs when corresponding human and mouse disorders are investigated in parallel.

## Materials and Methods

### DGAP individuals studied.

*DGAP104.* DGAP104 is the product of in vitro fertilization via intracytoplasmic sperm injection, whose parents of European descent were unrelated with no reported medical problems. Amniocentesis demonstrated a 46,XX,t(1;20)(p32.3;q13.31)dn, but ultrasound revealed no organ malformations at 20 wk of pregnancy. Because of placenta previa and persistent vaginal bleeding, DGAP104 was delivered at 31 wk via elective cesarean section and weighed 1,980 g with Apgar scores of 9/10/10. She was diagnosed with prematurity, hydrocephaly, Chiari I malformation, tethered spinal cord, congenital hydronephrosis, left hypoplastic kidney, bilateral inguinal hernia, hyaline membrane disease grade 2, and gastroesophageal reflux. Imaging studies indicated a thin posterior corpus callosum and an open aqueduct with progressive ventricular enlargement. She was delayed in reaching developmental milestones; at 2 y, she exhibited major motor delay with inhibited movement and was wheelchair bound. Speech was also delayed, and limited to a few words. At 6 y 7 mo of age, DGAP104 received a performance IQ score of 42, verbal IQ score of 68, and global IQ score of 52 on the Weschler Preschool and Primary Scale of Intelligence-Revised test (WPPSI-R).

To relieve hydrocephalus, which caused progressive macrocephaly, seven neurosurgical operations were performed over 5 y, including a third ventriculocisternostomy, a ventriculoperitoneal shunt with a flow-regulated valve, and several revisions because of hyperdrainage or blockage of the shunt. At 7 d of age, abdominal ultrasonography revealed bilateral hypoplastic kidneys and bilateral dilatation of the renal pelvis. At 1 y, ultrasound showed left and right kidney lengths of 45 mm and 51 mm, respectively (mean length for age, 52 mm), and both kidneys lacked corticomedullary differentiation. At 2 y, the left kidney length was 49 mm and the right 58 mm (mean length for age, 55 mm), but both lacked discernable corticomedullary differentiation. At 5 y, the left kidney was 53 mm and the right 61 mm (mean length for age, 66 mm). DGAP104 first exhibited left grade II VUR by VCUG at 1 y. At 2 y, VUR increased to grade III with a right vesicoureteral junction diverticulum, and pyelonephritis developed that required ureteral reimplantation surgery (Cohen operation). Urea and creatinine levels were normal at 5 y of age.

*DGAP089.* DGAP089 is a male with an interstitial deletion on 2q and a balanced translocation involving 1p and 2q. Additional clinical data are summarized in Table S1 and described in more detail elsewhere [22]. Seizure onset occurred at 19 mo, with treatment until age 6 y and no recurrences thereafter. He rolled to one side at 10 mo, to either side at 14 mo, and sat at 14 mo. At 3 y, he received the Bayley Scales of Infant Development test (2nd edition, BSID-II), and earned a Mental Developmental Index (MDI) that fell below the standard score of 50 with an age equivalent of 4 mo. He also received the Vineland Adaptive Behavior Scales test (parent form VABS), and obtained an adaptive behavior composite score of 45 corresponding to an age equivalent of 7 mo. He cruised at 4 y, and walked at 5 y. DGAP089 underwent a brain MRI at 6.5 y that revealed a hypoplastic corpus callosum with enlarged frontal horns, parallel in configuration, without evidence of increased intracranial pressure. The MRI also showed polymicrogyria and an 8-mm nodule along the lateral wall of the left frontal horn that likely represented a gray matter heterotopia. Renal ultrasound performed at 6.5 y was normal. There was no significant change in the physical examination and no focal sensorimotor deficits were noted. When last examined at age 8 y (2005), DGAP089 was no longer wearing leg braces, was able to walk and run on his toes, and could eat table foods without difficulty, but was not toilet trained. Speech consisted of about three words in appropriate context, and he was being taught American Sign Language (ASL). He could gesture, but was unable to follow commands, and engaged in self-stimulatory behavior.

*DGAP174.* DGAP174 was born to a 20-y-old mother at 37 wk by cesarean section and weighed 2,770 g (95th percentile) with length 49.5 cm (25th percentile) and occipitofrontal circumference 37.5 cm (75th percentile). Pregnancy was uncomplicated by teratogenic exposures or maternal illness. A second trimester prenatal ultrasound showed agenesis of the corpus callosum and enlarged ventricles. A CT scan of the brain in the immediate postnatal period confirmed the prenatal findings, and revealed ventriculomegaly with parallel lateral ventricles representing longitudinal bundles of Probst, and a tethered spinal cord. A small ventricular septal defect was also noted at birth. Chromosome analysis of peripheral blood lymphocytes revealed 46,XY,t(1;3)(p22;q21)dn. The neonate was discharged and seen again at 13 d of age in the genetics clinic. He was found to have metopic stenosis and bitemporal narrowing that was surgically corrected at 5 mo of age. Neurological exam revealed normal tone and reflexes. At 8 mo of age, he was noted to be gaining weight rapidly, unrelated to any change in eating pattern. Follow-up at 12 mo revealed delay in gross motor development, macrocephaly without hydrocephalus, and height, weight, and length above the 95th percentile for his age. Additionally, a dimple on the posterior aspect of the right helix, creases behind each earlobe, and esotropia secondary to telecanthus and epicanthal folds were noted. A left inguinal hernia was detected at 24 mo. Also at 24 mo, glasses were prescribed to correct hypertropia and strabismus, and all milestones were on track except for speech. At 36 mo, expressive language was still delayed. Due to macrosomia, the patient underwent bone age testing, which was age appropriate. A brain MRI at 36 mo of age revealed Chiari I malformation and dysplasia of the anterior aspect of the left temporal fossa in addition to complete agenesis of the corpus callosum. At 57 mo of age, DGAP174 underwent several developmental tests. His IQ score was 68 on a Leiter International Performance Scale (LIPS) test, corresponding to an age equivalent of 36 mo. He received a standard score of 60 in the Peabody Picture Vocabulary Test (Revised, Form M; PPVT-R) corresponding to an age equivalent of 35 mo. His standard score for the Developmental Test of Visual-Motor Integration (VMI) was 67, which also corresponded to an age equivalent of 35 mo. At 6 y of age, he underwent a successful Chiari decompression and repair of the tethered spinal cord. At that time, he was diagnosed with attention deficit and hyperactivity disorder and is currently on Ritalin. He was noted to have right hemihypertrophy and scoliosis, for which he was referred to an orthopedics clinic. At age 8 y and 4 mo (January 2007), DGAP174 was functioning at the kindergarten level, and received occupational and physical therapy for speech.

*DGAP205–1, 205–1s,* and *205–2.* DGAP205–1 and DGAP205-1s are two half-siblings with an interstitial microdeletion, del(1)(p31.3p32.3), that was inherited as an unbalanced segregant resulting from a balanced rearrangement in their mother, DGAP205–2. Both half-siblings had congenital CNS and urinary tract defects while their mother was phenotypically normal. DGAP205–1 had the Bayley Scales of Infant Development test (BSID) at 4.5 y of age and received scores corresponding to an age equivalent of 18 mo for overall development, 20 mo for cognition, and 17 mo for fine motor. At age 5 y, DGAP205–1 was functioning at an age equivalent of ~2.5 y. At age 10 y he was functioning at an age equivalent of ~7 y, and was 4 ft tall and weighed ~65 lb. DGAP205–1 has limited verbal skills and uses a combination of words and signs for communication. He has not had any formal testing at 10 y of age, but can only read and spell three-letter words. DGAP205-1s received the Bayley Scales of Infant Development test (BSID) at 2 y of age, and the Stanford-Binet test at 9 y. Both tests demonstrated moderate global cognitive impairment (scores not available). At 9 y of age, she was functioning academically at a kindergarten level. Other clinical data for the affected sibs are summarized in Table S1, and described in more detail elsewhere [23].

### FISH, aCGH, and mutation screening.

Metaphase FISH was performed according to standard methods. BAC clones were obtained from BAC/PAC Resources (http://bacpac.chori.org), labeled as FISH probes, and hybridized to metaphase chromosomes prepared from lymphoblastoid cell lines established from all five individuals. The full BAC names provided in Figure 2I are: RP4-654H19, RP11-55M23, RP5-829C20, RP11-13N22, RP4-802A10, RP4-662P1, RP5-1078M7, RP4-534K7, RP11-86K19, RP11-131O15, and RP11-89K2. aCGH experiments were performed with the Spectral Genomics 2600 BAC array by the Cytogenetics Core Facility of the Dana-Farber/Harvard Cancer Center for DGAP089 and DGAP174, and by Spectral Genomics for DGAP104. *NFIA* mutation screening employed PCR amplification of the 11 human *NFIA* exons and intron-exon boundaries, followed by purification and bidirectional DNA sequencing. *NFIA* cDNA sequence AB037860 (http://www.ncbi.nlm.nih.gov/entrez/viewer.fcgi?db=nucleotide&val=7243275) was used to calculate nucleotide positions. MLPA analysis of the *NFIA* coding region was performed in a subset of syndromic and nonsyndromic ACC samples, and no copy number changes were identified.

### RT-PCR analysis.

RT-PCR analyses were performed by routine protocols. RT-PCR primers used to amplify the *Nfia* 334 bp cDNA were m*Nfia-rt-F* (5′-CAAGCCTCCAACCACATCAAC-3′) and m*Nfia-rt-R* (5′-CTGTTTGACCACGATGTTTGCT-3′). RT-PCR primers used to amplify the *C20orf32* 436 bp cDNA were m*C20orf32*-F (5′-GGGCACTCTACGACAACCAT-3′) and m*C20orf32*-R: (5′-TCTGGGAAGCACAGAGAGG-3′).

### Southern and nothern blot analysis.

Southern blotting was performed by standard methods. Probes were labeled using the MegaPrime labeling kit (Amersham/GE Healthcare, http://www.amershamhealth-us.com). Genomic DNA from the DGAP089 cell line and from a karyotypically normal control were digested with DraII, PstI, and SspI and hybridized with a 700-bp probe ampli-fied from RP5-902P15. This probe (AL096888; 66436–67143) was amplified by the following primers: forward primer: 5′-CAGGCTTCTTCCCTCACAAG3′ and reverse primer: 5′-GGTCCTTTCACGTGCATCTT-3′. Southern blots of BpmI, BspHI, DraII, EcoRI, HindIII, PvuII, and XhoI-digested DNA from DGAP104 and genomic DNA from a karyotypically normal male control were hybridized with a 623-bp probe that was amplified from RP4-802A10 (AC096947.2; 50191–50687). This probe was amplified by the following primers: forward primer: 5′-AGGCACCAGGGCAGTAATC-3′ and reverse primer: 5′-TAAGAACTCCAACCCCAGCA-3′. A northern blot containing poly A+ RNA from multiple regions of human brain (Human Brain V blot, Clontech, http://www.clontech.com) was hybridized with a probe corresponding to exons 2–6 of *NFIA* following a standard protocol.

### Analysis of *Nfia* mutant mice.

The generation and analysis of brain defects in *Nfia* knockout mice in a C57BL/6 background has been previously described [18]. *Nfia* ^−/−^ mice analyzed in Figure S6 were C57BL/6X129S6 *F*_1_ hybrid anaimals that have longer postnatal survival than C57BL/6 inbred mice. The *Nfia* ^−^ allele was genotyped by PCR amplification using the mutant allele specific forward primer *Nfia*-ln1-F2 (5′-CGTGAGCTGGTACAGTTTGCA-3′, in the non-deleted region of *Nfia* intron 1) and reverse primer *Nfia*-Neo-R2 (5′-GCCTGAAGAACGAGATCAGCA-3′, in the neomycin selection cassette). The *Nfia* wild-type allele was genotyped using the same forward primer *Nfia*-ln1-F2 and a gene-specific reverse primer *Nfia*-WT-R2 (5′-TGTTTGAGAATCTTTCTTCCTTTGG-3′, in the deleted region of *Nfia* intron 1). Histological analyses were performed on mouse specimens fixed in 4% paraformaldehyde, embedded in paraffin, sectioned at 4 μm, and stained with hematoxylin and eosin. To examine ureter and kidney defects, *Hoxb7-GFP* transgenic mice (gift from Dr. Frank Costantini, Columbia University) were bred with *Nfia* knockout mutants. GFP fluorescence illumination of the mouse urinary tract was evaluated using a Nikon SMZ-1500 epi-fluorescence stereomicroscope (http://www.nikonusa.com).

### In situ hybridization and immunohistochemistry.

Tissue in situ hybridization of whole mount and cryosections was performed according to standard protocols using cRNA probes complementary to the 3′-UTRs of *Nfia* and *C20orf32*. For WT1 immunostaining, kidney samples were fixed in 4% paraformaldehyde for 30 min and embedded in OCT Compound. Tissues were then cryosectioned at 10 μm and stained with anti-WT1 antibody (Santa Cruz Biotechnology, http://www.scbt.com). Dolichos Biflorus Agglutinin (DBA; Vector Labs, http://www.vectorlabs.com) and *Lotus Tetragonolobus* Lectin (LTL; Vector Labs) stainings were performed on paraffin-embedded kidney sections.

### Ethics.

All human studies were performed under informed consent protocols approved by the Human Research Committee of Partners HealthCare System, Boston. Mouse protocols were approved by the Institutional Animal Care and Use Committee at Harvard Medical School or at Boston University Medical Center.

## Supporting Information

Figure S1 Southern Blot Analysis of 1p31.3 Breakpoint in DGAP104 *NFIA* is disrupted in DGAP104 and the breakpoint lies within intron 2.
(A) Southern blot analysis of DGAP104 (P) and normal control (C) genomic DNA using the designated restriction enzymes and the probe A4 shown in panel B. Aberrant bands (arrows in A) are present only in DGAP104 DNA digested with BpmI, BspHI, DraII, EcoRI, and XhoI.
(B) Restriction map surrounding the *NFIA* intron 2 region. The base-pair position of BAC RP4-802A10 (AC096947, within intron 2 of *NFIA*, see BAC contig in Fig. 2I) was used to calculate the distance between restriction enzyme sites. BAC RP4-802A10 was used in FISH and contains the breakpoint, which is between boxed PvuII and BpmI sites based on the aberrant bands detected by Southern blot analysis.
(167 KB PDF)

Figure S2 In Situ Hybridization of Mouse *C20orf32*
*C20orf32* is not expressed in the mouse embryonic spinal cord and kidney at E10.5 and E11.5.
(33 KB PDF)

Figure S3 Southern Blot Analysis of 1p31.3 Breakpoint in DGAP089 *NFIA* is disrupted in DGAP089 and the breakpoint lies within intron 7.
(A) Southern blot analysis of DGAP089 (P) and normal control (C) genomic DNA using the designated restriction enzymes and the probe A1 shown in panel B. Aberrant bands (arrows in A) are present in DGAP089 DNA digested with DraII, PstI and SspI.
(B) Restriction map surrounding the *NFIA* intron 7 region. The base-pair position of BAC RP5-902P15 (AL096888, within intron 7 of *NFIA*, see BAC contig in Fig. 2I) was used to calculate the distance between restriction enzyme sites. BAC RP5-902P15 was used in FISH and contains the breakpoint, which is between boxed SspI and DraII sites based on the aberrant bands detected by Southern blot analysis.
(104 KB PDF)

Figure S4 Array Comparative Genomic Hybridization (aCGH) for DGAP174 Array CGH at a 1-Mb resolution defines the deletion interval in DGAP174. Spectral Genomics profile indicates the deletion interval (arrow) on 1p from 1p32.1 to 1p31.3.
(131 KB PDF)

Figure S5 Northern Blot Analysis of *NFIA* in Different Regions of the Human Brain Northern blot of multiple human brain tissues was hybridized with a probe containing *NFIA* exons 2–6.
(109 KB PDF)

Figure S6 Hydronephrosis in P16 *Nfia* ^−/−^ Mutant Severe hydronephrosis (* in B) is shown in a P16 *Nfia* ^−/−^ mutant kidney, whereas the kidney in its wild-type littermate (A) is normal.
(94 KB PDF)

Table S1 Clinical Findings in Five Individuals with Chromosome Abnormalities Involving 1p31 (61 KB DOC)

Table S2 Phenotypes of 84 Patients with Callosal and other CNS Malformations and Urinary Tract Defects Subjected to *NFIA* Intragenic Mutation Screening (144 KB DOC)

### Accession Numbers

The GeneID numbers for the Entrez Genes (http://www.ncbi.nlm.nih.gov/entrez/query.fcgi?db=gene) discussed in this paper are: *AK3L2* (387851), *ALG6* (29929), *ANGPTL3* (27329), *ANKRD38* (163782), *ATG4C* (84938), *BSND* (7809), *C1orf87* (127795), *C1orf141* (400757), *C1orf168* (199920), *C8A* (731), *C8B* (732), *C20orf32* (57091), *C20orf32* (320664), *CACHD1* (57685), *CYP2J2* (1573), *DAB1* (1600), *DNAJC6* (9829), *DOCK7* (85440), *FLJ10986* (55277), *FLJ45337* (400754), *FOXD3* (27022), *HOOK1* (51361), *IL23R* (149233), *INADL* (10207), *INSL5* (10022), *ITGB3BP* (23421), *JAK1* (3716), *JUN* (3725), *KIAA1799* (84455), *L1TD1* (54596), *LEPR* (3953), *LEPROT* (54741), *MIER1* (57708), *NEGR1* (257194), *NFIA* (4774), *Nfia* (18027), *OMA1* (115209), *PCSK9* (255738), *PDE4B* (5142), *PGM1* (5236), *PPAP2B* (8613), *PRKAA2* (5563), *RAVER2* (55225), *ROR1* (4919), *SGIP1* (84251), *SLC35D1* (23169), *TACSTD2* (4070), *TCTEX1D1* (200132), *TM2D1* (83941), *UBE2U* (148581), *USP1* (7398), and *WDR78* (79819).

The disease identifiers for the OMIM (http://www.ncbi.nlm.nih.gov/entrez/query.fcgi?db=OMIM) genetic disorders discussed in this paper are: agenesis of the corpus callosum (OMIM 217990), hydrocephalus and ventriculomegaly (OMIM 236600), Chiari malformation type I (OMIM 118420), and vesicoureteral reflux (OMIM 193000 and 610878).

## Abbreviations

ACC: agenesis of the corpus callosum
aCGH: array comparative genomic hybridization
BAC: bacterial artificial chromosome
CNS: central nervous system
FISH: fluorescence in situ hybridization
OMIM: Online Mendelian Inheritance in Man
UPJ: ureteropelvic junction
UVJ: ureterovesical junction
VCUG: voiding cystourethrogram
VUR: vesicoureteral reflux
