# Supplementary material for: NFIA Haploinsufficiency Is Associated with a CNS Malformation Syndrome and Urinary Tract Defects
Source: PLoS Genet. 2007 May 25;3(5):e80. doi: 10.1371/journal.pgen.0030080 (PMC1877820; doi:10.1371/journal.pgen.0030080)
Supplement: Figure S1 — NFIA is disrupted in DGAP104 and the breakpoint lies within intron 2. (A) Southern blot analysis of DGAP104 (P) and normal control (C) genomic DNA using the designated restriction enzymes and the probe A4 shown in panel B. Aberrant bands (arrows in A) are present only in DGAP104 DNA digested with BpmI, BspHI, DraII, EcoRI, and XhoI. (B) Restriction map surrounding the NFIA intron 2 region. The base-pair position of BAC RP4-802A10 (AC096947, within intron 2 of NFIA, see BAC contig in Fig. 2I) was used to calculate the distance between restriction enzyme sites. BAC RP4-802A10 was used in FISH and contains the breakpoint, which is between boxed PvuII and BpmI sites based on the aberrant bands detected by Southern blot analysis. (167 KB PDF) [file pgen.0030080.sg001.pdf]

Supporting information

Figure S1. Southern blot analysis of 1p31.3 breakpoint in DGAP104

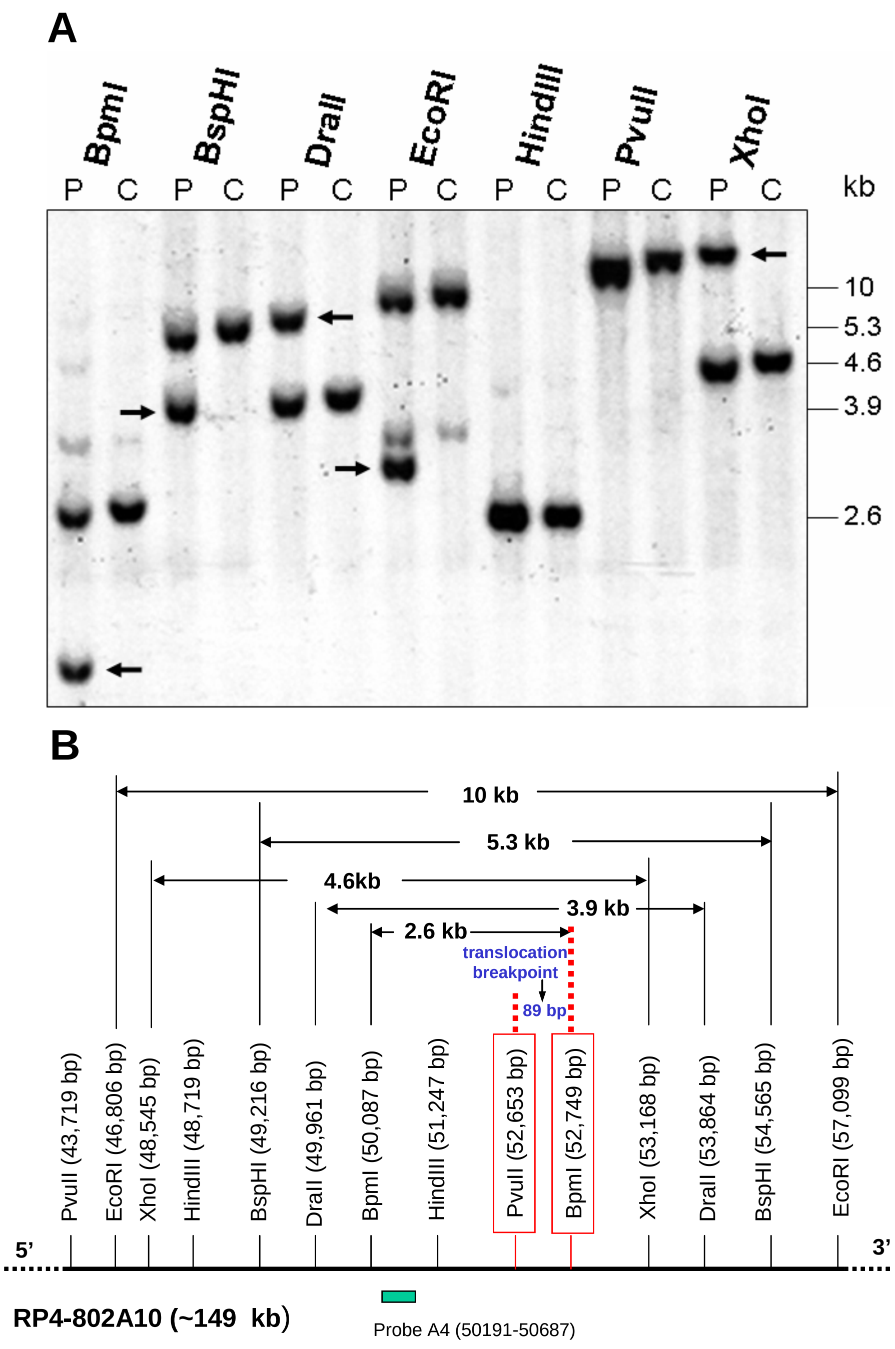

***NFIA* is disrupted in DGAP104 and the breakpoint lies within intron 2. (A)** Southern blot analysis of DGAP104 (P) and normal control (C) genomic DNA using the designated restriction enzymes and the probe A4 shown in panel **B.** Aberrant bands (arrows in **A**) are present only in DGAP104 DNA digested with *Bpm*I, *BspH*I, *Dra*II, *EcoR*I and *Xho*I. (**B**) Restriction map surrounding the *NFIA* intron 2 region. The base pair position of BAC RP4-802A10 (AC096947, within intron 2 of *NFIA*, see BAC contig in Fig. 1I) was used to calculate the distance between restriction enzyme sites. BAC RP4-802A10 was used in FISH and contains the breakpoint, which is between boxed *Pvu*II and *Bpm*I sites based on the aberrant bands detected by Southern blot analysis.
