# Supplementary material for: NFIA Haploinsufficiency Is Associated with a CNS Malformation Syndrome and Urinary Tract Defects
Source: PLoS Genet. 2007 May 25;3(5):e80. doi: 10.1371/journal.pgen.0030080 (PMC1877820; doi:10.1371/journal.pgen.0030080)
Supplement: Figure S2 — C20orf32 is not expressed in the mouse embryonic spinal cord and kidney at E10.5 and E11.5. (33 KB PDF) [file pgen.0030080.sg002.pdf]

Figure S2. ***In situ*** **hybridization of mouse** ***C20orf32***

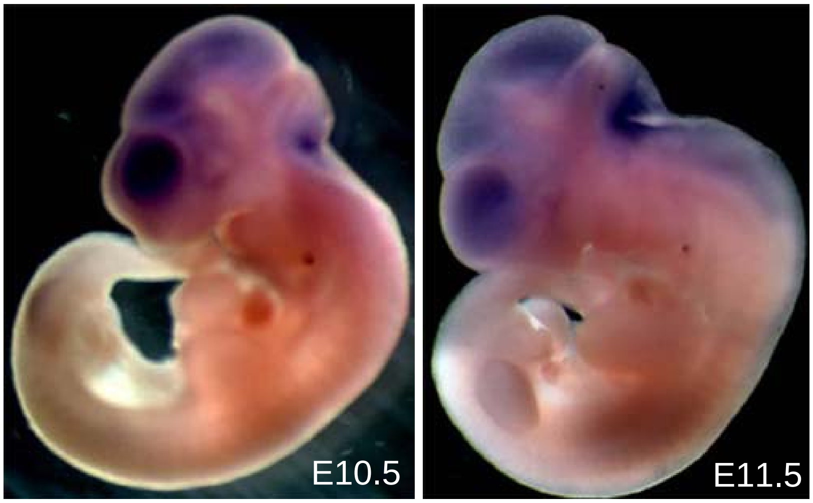

C20orf32 is not expressed in the mouse embryonic spinal cord and kidney at E10.5 and E11.5.
