# Supplementary material for: NFIA Haploinsufficiency Is Associated with a CNS Malformation Syndrome and Urinary Tract Defects
Source: PLoS Genet. 2007 May 25;3(5):e80. doi: 10.1371/journal.pgen.0030080 (PMC1877820; doi:10.1371/journal.pgen.0030080)
Supplement: Figure S3 — NFIA is disrupted in DGAP089 and the breakpoint lies within intron 7. (A) Southern blot analysis of DGAP089 (P) and normal control (C) genomic DNA using the designated restriction enzymes and the probe A1 shown in panel B. Aberrant bands (arrows in A) are present in DGAP089 DNA digested with DraII, PstI and SspI. (B) Restriction map surrounding the NFIA intron 7 region. The base-pair position of BAC RP5-902P15 (AL096888, within intron 7 of NFIA, see BAC contig in Fig. 2I) was used to calculate the distance between restriction enzyme sites. BAC RP5-902P15 was used in FISH and contains the breakpoint, which is between boxed SspI and DraII sites based on the aberrant bands detected by Southern blot analysis. (104 KB PDF) [file pgen.0030080.sg003.pdf]

Figure S3. **Southern blot analysis of 1p31.3 breakpoint in DGAP089**

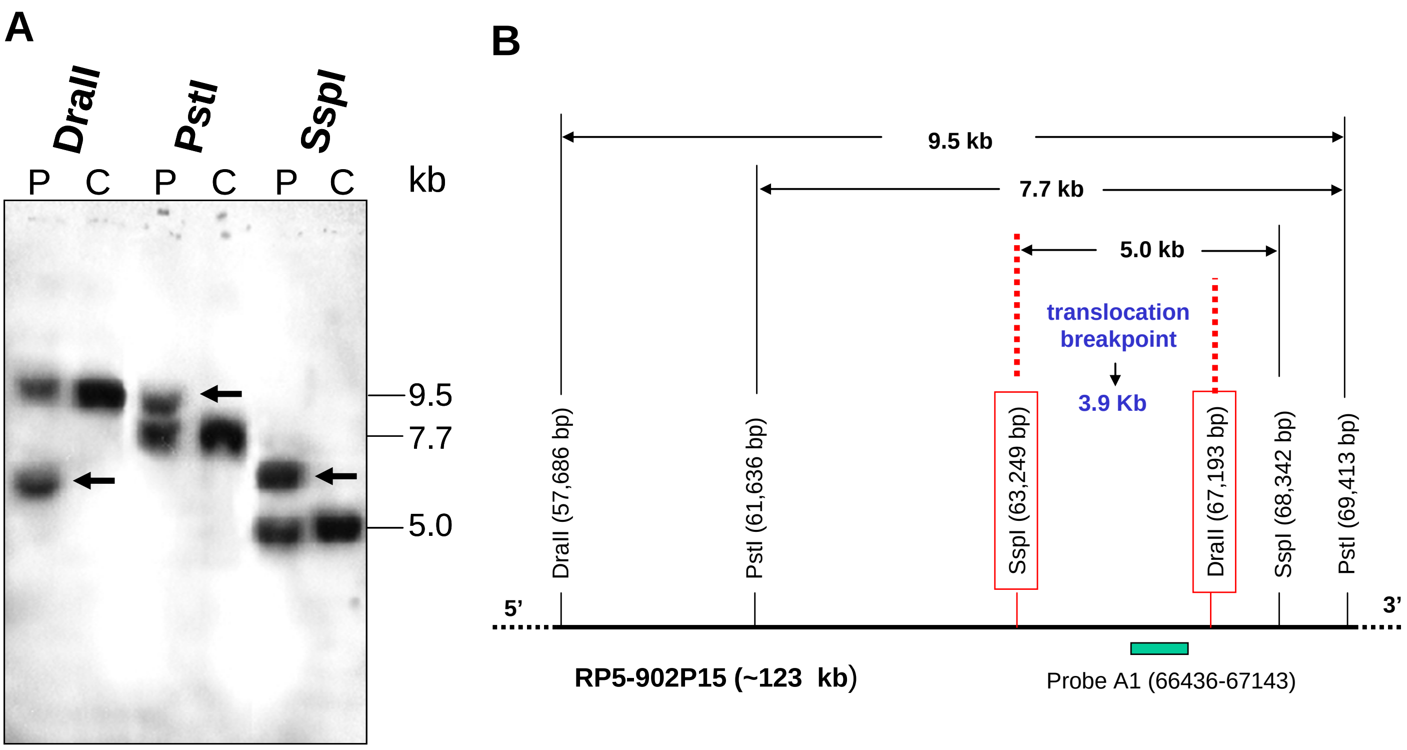

***NFIA* is disrupted in DGAP089 and the breakpoint lies within intron 7.** **(A)** Southern blot analysis of DGAP089 (P) and normal control (C) genomic DNA using the designated restriction enzymes and the probe A1 shown in panel **B.** Aberrant bands (arrows in **A**) are present in DGAP089 DNA digested with *Dra*II, *Pst*I and *Ssp*I. **(B)** Restriction map surrounding the *NFIA* intron 7 region. The base pair position of BAC RP5-902P15 (AL096888, within intron 7 of *NFIA*, see BAC contig in Fig. 1I) was used to calculate the distance between restriction enzyme sites. BAC RP5-902P15 was used in FISH and contains the breakpoint, which is between boxed *Ssp*I and *Dra*II sites based on the aberrant bands detected by Southern blot analysis.
