# Supplementary material for: NFIA Haploinsufficiency Is Associated with a CNS Malformation Syndrome and Urinary Tract Defects
Source: PLoS Genet. 2007 May 25;3(5):e80. doi: 10.1371/journal.pgen.0030080 (PMC1877820; doi:10.1371/journal.pgen.0030080)
Supplement: Figure S4 — Array CGH at a 1-Mb resolution defines the deletion interval in DGAP174. Spectral Genomics profile indicates the deletion interval (arrow) on 1p from 1p32.1 to 1p31.3. (131 KB PDF) [file pgen.0030080.sg004.pdf]

Figure S4. **Array Comparative Genomic Hybridization (aCGH) for DGAP174**

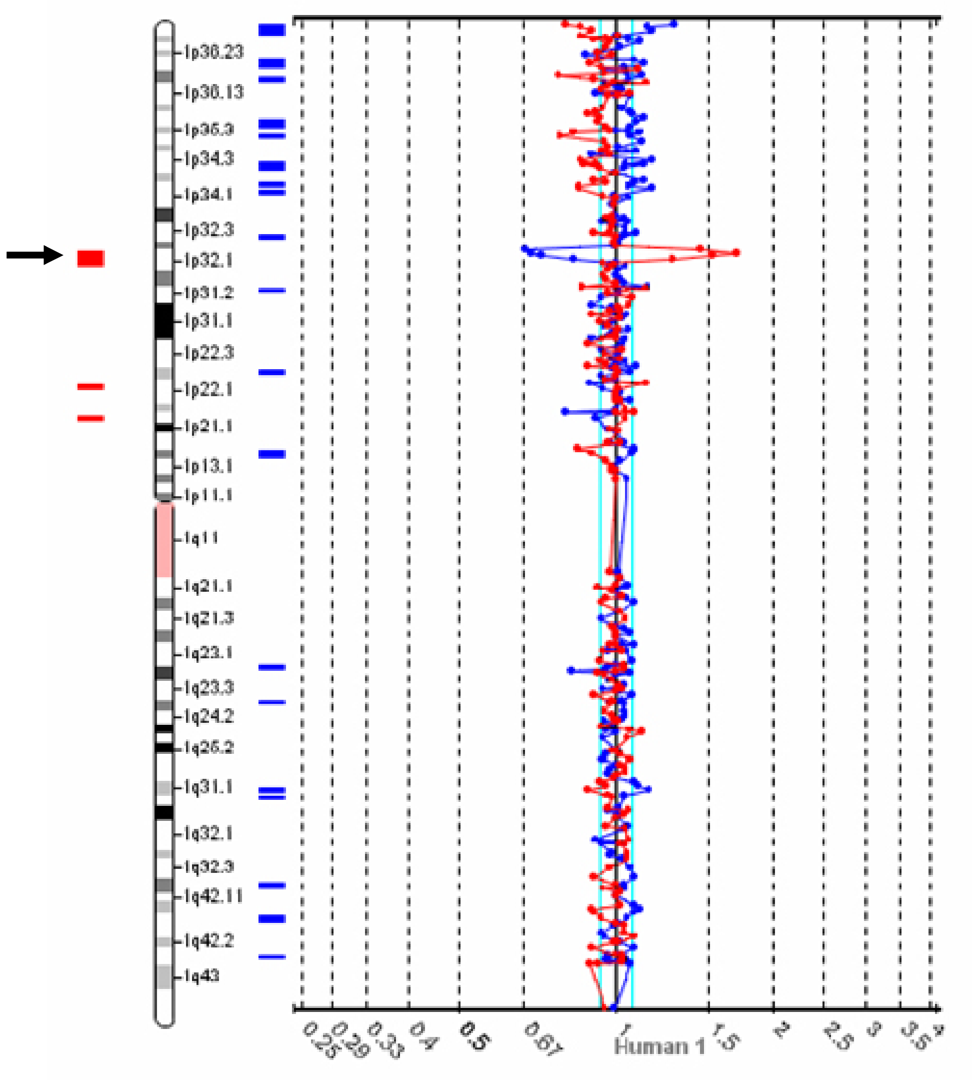

Array CGH at a 1-Mb resolution defines the deletion interval in DGAP174. Spectral Genomics profile indicates the deletion interval (arrow) on 1p from 1p32.1 to 1p31.3.
