# Supplementary material for: NFIA Haploinsufficiency Is Associated with a CNS Malformation Syndrome and Urinary Tract Defects
Source: PLoS Genet. 2007 May 25;3(5):e80. doi: 10.1371/journal.pgen.0030080 (PMC1877820; doi:10.1371/journal.pgen.0030080)
Supplement: Figure S5 — Northern blot of multiple human brain tissues was hybridized with a probe containing NFIA exons 2–6. (109 KB PDF) [file pgen.0030080.sg005.pdf]

Figure S5. **Northern blot analysis of *NFIA* in different regions of the human brain**

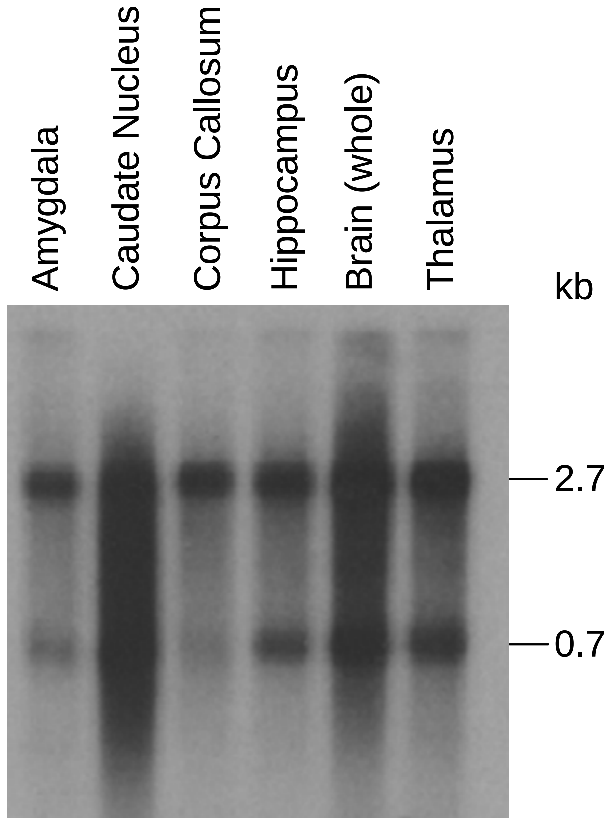

Northern blot of multiple human brain tissues was hybridized with a probe containing *NFIA* exons 2-6.
