# Supplementary material for: NFIA Haploinsufficiency Is Associated with a CNS Malformation Syndrome and Urinary Tract Defects
Source: PLoS Genet. 2007 May 25;3(5):e80. doi: 10.1371/journal.pgen.0030080 (PMC1877820; doi:10.1371/journal.pgen.0030080)
Supplement: Figure S6 — Severe hydronephrosis (* in B) is shown in a P16 Nfia −/− mutant kidney, whereas the kidney in its wild-type littermate (A) is normal. (94 KB PDF) [file pgen.0030080.sg006.pdf]

Figure S6. **Hydronephrosis in P16 *Nfia*$^{-/-}$ mutant**

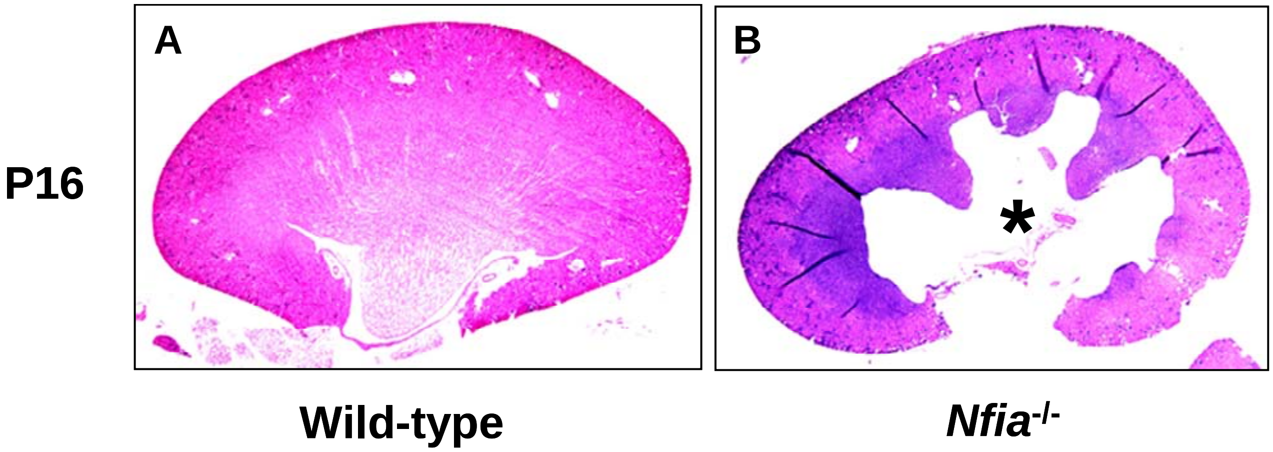

Severe hydronephrosis (* in B) is shown in a P16 *Nfia*$^{-/-}$ mutant kidney, whereas the kidney in its wild-type littermate (A) is normal.
