# Supplementary material for: NFIA Haploinsufficiency Is Associated with a CNS Malformation Syndrome and Urinary Tract Defects
Source: PLoS Genet. 2007 May 25;3(5):e80. doi: 10.1371/journal.pgen.0030080 (PMC1877820; doi:10.1371/journal.pgen.0030080)
Supplement: Table S1 — (61 KB DOC) [file pgen.0030080.st001.doc]

**Table S1**. Clinical findings in five individuals with chromosome abnormalities involving 1p31

| **Clinical Characteristic** | **DGAP089** | **DGAP104** | **DGAP174** | **DGAP205-1** | **DGAP205-1s** |
| --- | --- | --- | --- | --- | --- |
| Brain abnormalities | Hypoplastic corpus callosum, polymicrogyria, gray matter heterotopia | Thin posterior corpus callosum | Complete agenesis of corpus callosum, dysplasia of left temporal fossa | Thin corpus callosum, decreased periventricular white matter | Absence of the corpus callosum, patchy lucencies in the frontal lobes |
| Ventricle defects | Non-progressive ventriculomegaly. | Congenital communicating hydrocephalus | Non-progressive enlarged ventricles | Enlarged ventricles, large anterior CSF spaces | Enlarged ventricles |
| Neural tube defects | Not detected | Tethered spinal cord, Chiari I malformation | Tethered spinal cord, Chiari I malformation | Tethered spinal cord | Tethered spinal cord, Chiari I malformation, syringomyelia |
| Urinary tract defects | Normal renal ultrasound at 6.5 years, no VCUG performed | Left vesicoureteral reflux grade III, right UVJ diverticulum, bilateral dysplastic kidney | Normal renal ultrasound, no VCUG performed | Bilateral vesicoureteral reflux, urosepsis | Urinary incontinence, urinary tract infection at 7 years |
| Skeleton abnormality | Craniofacial disproportion and relative macrocephaly | Fishhook deformation of lower sacral and coccygeal vertebrae | Macrocephaly, metopic stenosis, bitemporal narrowing | Right thumb polydactyly, left foot varus deformity | Congenital hip dysplasia |
| Development | Product of a full term pregnancy with 2,800g at birth, gross motor, receptive and expressive language delay. >50 on BSID-II test score and 45 VABS test score at 3 years | Premature birth at 31 weeks, motor and verbal retardation. Performance IQ 42, verbal IQ 68, and global IQ 52 at 6.5 years | Product of a 37 weeks pregnancy. Received 68 score on LIPS, 60 on PCT-R, and 67 on VMI at 57 months. Speech delay at 8 years | Product of a normal pregnancy, moderate motor and verbal retardation. Can read and spell 3 letter words at 10 years | Product of a normal 41 weeks gestation. BSID test at 2 years and Stanford Binet test at 9 years showed global.cognitive impairment. |
| Eye defects | Not detected | Pigmentary retinopathy | Hypertropia, strabismus | Canthal hypertelorism | Up-slanting palpebral fissures |
| Other abnormalities | Seizures, cryptorchidism, chronic intestinal obstruction | Seizures, bilateral inguinal hernia, hypotonia | Left inguinal hernia, ADHD, hypotonia | Seizures, right inguinal hernia, cryptorchidism, cholelithiasis, right hemiplegia | Developed severe respiratory distress at 13 years, died of RSV pneumonia |
