# Supplementary material for: NFIA Haploinsufficiency Is Associated with a CNS Malformation Syndrome and Urinary Tract Defects
Source: PLoS Genet. 2007 May 25;3(5):e80. doi: 10.1371/journal.pgen.0030080 (PMC1877820; doi:10.1371/journal.pgen.0030080)
Supplement: Table S2 — (144 KB DOC) [file pgen.0030080.st002.doc]

**Table S2.** Phenotypes of 84 patients with callosal and other CNS malformations and urinary tract defects subjected to *NFIA* intragenic mutation screening

| **Patient ID** | **CNS and urinary tract phenotype** |
| --- | --- |
| AGB-43 | hypoplastic CC, MM, hydrocephalus |
| AGB-44 | thin CC, MM, hydrocephalus, solitary right kidney |
| AGB-45 | hypoplastic CC, MM, hydrocephalus, solitary right kidney |
| AGB-46 | thin CC, hydrocephalus, MM |
| AGB-47 | abnormally shaped CC, MM, hydrocephalus |
| AGB-48 | abnormally shaped CC, MM, hydrocephalus |
| AGB-49 | abnormally shaped CC, MM, hydrocephalus |
| AGB-50 | hypoplastic CC, MM, hydrocephalus |
| AGB-51 | hypoplastic, abnormally shaped CC, MM, hydrocephalus, small right kidney, neurogenic bladder |
| AGB-52 | abnormally shaped CC, MM, hydrocephalus |
| AGB-53 | thin CC, MM, hydrocephalus |
| AGB-54 | thin CC, MM, hydrocephalus |
| AGB-55 | thin CC, MM, hydrocephalus |
| AGB-56 | thin CC, hydrocephalus, MM |
| AGB-57 | thin CC, MM, hydrocephalus |
| AGB-58 | hypoplastic CC, hydrocephalus, MM |
| AGB-59 | very abnormal CC, MM, hydrocephalus |
| AGB-60 | questionably thin CC, tethered spinal cord, bilateral hydronephrosis |
| AGB-61 | hypoplastic CC, MM, hydrocephalus |
| AGB-62 | very abnormal CC, MM, hydrocephalus |
| AGB-63 | abnormally shaped CC, MM, hydrocephalus |
| AGB-64 | thin CC, MM, hydrocephalus |
| AGB-65 | abnormally shaped CC, MM, hydrocephalus |
| AGB-66 | thin CC, MM, hydrocephalus |
| AGB-67 | thin CC, MM, hydrocephalus |
| AGB-68 | thin CC, MM, hydrocephalus |
| AGB-69 | hypoplastic CC, MM, hydrocephalus |
| AGB-70 | hypoplastic CC, MM, hydrocephalus |
| AGB-71 | hypoplastic CC, MM, hydrocephalus |
| AGB-72 | thin CC, MM, hydrocephalus |
| AGB-73 | very abnormal CC, MM, hydrocephalus |
| AGB-74 | thin CC, MM, hydrocephalus |
| AGB-75 | thin CC, hydrocephalus, MM, small right kidney |
| AGB-76 | questionably thin CC, MM, hydrocephalus |
| AGB-77 | abnormally shaped CC, tethered spinal cord |
| AGB-78 | questionably thin CC, tethered spinal cord |
| AGB-79 | thin CC, MM, hydrocephalus |
| AGB-80 | MM, very abnormal CC, bilateral hydronephrosis, mild dilatation of right ureter |
| AGB-81 | MM, abnormal CC |
| AGB-82 | very abnormal CC, MM, hydrocephalus, left hydronephrosis, neurogenic bladder |
| AGB-83 | very thin CC, MM, hydrocephalus |
| AGB-84 | abnormally shaped hypoplastic CC |
| AGB-85 | thin CC, hydrocephalus, MM |
| AGB-86 | normal CC, tethered spinal cord |
| AGB-87 | very thin CC, normal spinal cord |
| AGB-88 | very thin CC, MM, hydrocephalus |
| AGB-89 | hypoplastic CC, MM, hydrocephalus |
| AGB-90 | hypoplastic CC, MM, hydrocephalus, solitary left kidney |
| AGB-91 | hypoplastic CC, MM, hydrocephalus |
| AGB-92 | mild hydrocephalus |
| AGB-93 | hydrocephalus, normal spine |
| AGB-94 | thin CC, hydrocephalus |
| AGB-95 | Chiari I malformation, normal CC |
| AGB-96 | Chiari I malformation, normal CC |
| AGB-97 | questionably thin CC, normal spine |
| AGB-98 | hypoplastic CC, tethered spinal cord |
| AGB-99 | normal CC, Chiari I malformation |
| AGB-100 | questionably thin CC, tethered spinal cord |
| AGB-101 | absence of the CC |
| AGB-102 | absence of the CC |
| AGB-103 | very abnormally shaped CC, mild hydrocephalus |
| AGB-104 | somewhat abnormally shaped CC, tethered spinal cord |
| AGB-105 | thin CC, normal spinal cord |
| AGB-106 | complete absence of the CC |
| AGB-107 | very abnormal CC, hydrocephalus, normal spine |
| AGB-108 | abnormally shaped CC, tethered spinal cord |
| AGB-109 | Chiari I malformation |
| AGB-110 | hypoplastic CC, MM, hydrocephalus |
| OTH-2501 | ACC and interhemispheric cyst |
| IHC-1301 | ACC with interhemispheric cyst Type Iic, subcortical heterotopia, enlarged trigones, white matter reduced in parietal/occipital lobes |
| IHC-1402 | ACC with interhemispherics cyst Type 1b, subependymal heterotopia, diminished white matter |
| IHC-1501 | ACC with interhemispheric cyst |
| IHC-501 | near complete ACC with only part of genus present, bilateral R>L bundles of Probst, interhemispheric cysts, posterior fossa abnl |
| MC-6301 | hypoplastic CC, reduced white matter, hypoplastic cerebellum, microcephaly |
| MC-6801 | frontal lobe hypoplasia; CC: only body is present, absent anterior commissure; white matter diffusely diminished; microcephaly |
| MC-7901 | hypoplastic body/splenium, agenesis of rostrum of CC, microcephaly |
| MC-9101 | ACC, subependymal nodular heterotopia, microcephaly, reduced white matter |
| AGB-111 | ACC, ventriculomegaly |
| AGB-112 | Ventriculomegaly, tethered spinal cord, neurogenic bladder |
| VB | ACC, enlarged ventricles, pontine atrophy, abnormal kidney corticomedullary differentiation |
| VDBL | Enlarged ventricular system, VUR |
| RA | Chiari 1 malformation, smaller right kidney |
| WK | Hydrocephaly, Dandy-Walker hypoplastic vermis and cerebellum, agenesis of left kidney, right kidney cyst |
| MS | ACC, enlarged occipital horns, VUR |

Abbreviations: CC, corpus callosum; ACC, agenesis of corpus callosum; MM, myelomeningocele;

VUR, vesicoureteral reflux
